# Different Resting State EEG Features in Children from Switzerland and Saudi Arabia

**DOI:** 10.3389/fnhum.2016.00559

**Published:** 2016-11-02

**Authors:** Nsreen Alahmadi, Sergey A. Evdokimov, Yury (Juri) Kropotov, Andreas M. Müller, Lutz Jäncke

**Affiliations:** ^1^Department of Special Education, Institute of Higher Education Studies, King Abdulaziz UniversityJeddah, Saudi Arabia; ^2^N.P. Bechtereva Institute of the Human Brain, Russian Academy of SciencesSt. Petersburg, Russia; ^3^Brain and Trauma Foundation GrisonsChur, Switzerland; ^4^Department of Neuropsychology, Psychological Institute, University of ZurichZurich, Switzerland

**Keywords:** intercultural neuroscience, EEG, resting state EEG, children, LORETA, group independent component analysis

## Abstract

**Background**: Cultural neuroscience is an emerging research field concerned with studying the influences of different cultures on brain anatomy and function. In this study, we examined whether different cultural or genetic influences might influence the resting state electroencephalogram (EEG) in young children (mean age 10 years) from Switzerland and Saudi Arabia.

**Methods**: Resting state EEG recordings were obtained from relatively large groups of healthy children (95 healthy Swiss children and 102 Saudi Arabian children). These EEG data were analyzed using group independent components analyses (gICA) and conventional analyses of spectral data, together with estimations of the underlying intracortical sources, using LORETA software.

**Results**: We identified many similarities, but also some substantial differences with respect to the resting state EEG data. For Swiss children, we found stronger delta band power values in mesial frontal areas and stronger power values in three out of four frequency bands in occipital areas. For Saudi Arabian children, we uncovered stronger alpha band power over the sensorimotor cortex. The additionally measured theta/beta ratio (TBR) was similar for Swiss and Saudi Arabian children.

**Conclusions**: The different EEG resting state features identified, are discussed in the context of different cultural experiences and possible genetic influences. In addition, we emphasize the importance of using appropriate EEG databases when comparing resting state EEG features between groups.

## Introduction

During the first 20 years of life, the human brain changes substantially, with pruning of unused synapses and myelination of long axons taking place from preschool age until late adolescence (Paus, 2005; Dubois et al., 2008; Paus et al., 2008). On a macroscopic level, brain maturation is characterized by different maturational trajectories, with sensory and sensorimotor areas maturing first, followed by the frontal, parietal and finally the temporal areas. A typical feature of this anatomical maturation trajectory is the increase in white matter volume and white matter integrity during puberty until adolescence (Giedd et al., 1999; Gogtay et al., 2004; Shaw et al., 2008; Raznahan et al., 2011). For several brain regions, brain maturation follows an inverted u-shaped trajectory, which is especially evident in the frontal cortex, where the volume increases until the age of 11–12.5 years, followed by a decrease. Brain maturation during childhood and adolescence is also reflected in typical resting state electroencephalogram (EEG) features of decreasing delta and theta band power with increasing age. Associated with this decrease in slow EEG oscillations, is an increase in posterior alpha band power (Ahn et al., 1980; John et al., 1980; Alvarez et al., 1987; Gasser et al., 1988; Harmony et al., 1988).

The major driving forces behind these maturation processes are the genetic influences that control human brain development from birth to adolescence. However, anatomical and neurophysiological brain maturation is not entirely driven by genetic influences; it is also modulated by several non-genetic factors. For example, it is a well-known fact that nutritional status, education, and social influences strongly influence brain development (Hackman and Farah, 2009). Using resting state EEG, several studies have shown greater power in the slower EEG frequencies in children reared under conditions of extreme deprivation or social isolation (Zubek et al., 1963; Gendreau et al., 1972; Marshall and Fox, 2004; Vanderwert et al., 2015). This EEG feature has also been observed in typically developing children who live in low-resource homes and families, or in impoverished environments (e.g., poor nutrition, unsanitary living conditions, less responsive or sensitive caregiving, or low Socioeconomic status (SES; Harmony et al., 1988; Otero, 1994, 1997; Otero et al., 2003; Kustubaeva, 2013).

These influences aside, experience, cognitive training and motor training all modulate brain development on different levels (Jäncke, 2009; Zatorre et al., 2012). For example, musicians with early musical training typically demonstrate the strongest anatomical alterations in brain areas known to be involved in controlling musical instruments (Imfeld et al., 2009). Longitudinal training studies in children have also uncovered neuroanatomical and neurophysiological changes as a consequence of training (Moreno and Besson, 2006; Hyde et al., 2009; Moreno et al., 2011). One theory regarding these changes is that when these plastic changes are implemented during childhood, they might persist into adolescence and adulthood, and not only shape further brain development, but also substantially influence future performance in the practiced task (Jäncke et al., 1997; Cheung et al., 2009; Meyer et al., 2011).

Since these non-genetic influences modulate the development of anatomical and neurophysiological features, it is conceivable that cultural influences might also exert an impact on brain development (Han and Northoff, 2008; Ames and Fiske, 2010). Conceptually, the examination of cultural differences is an important methodology for understanding neuroplasticity, particularly when individuals from strongly differing cultures are examined. Different cultures are very often associated with considerably different influences on development, particularly when the cultures strongly differ. For example, Arabian and Western cultures provide developing children with considerably different social and environmental information, including different values, beliefs, and practices, which might have a substantial impact on brain development.

However, children from different ethnicities also differ in terms of particular genetic underpinnings. In fact, a seminal study from a large human genome analysis revealed the striking genetic similarity displayed by people living in the Middle-East, including Arab countries (Li et al., 2008). Since it is known that resting state EEG is strongly influenced by genetics (Smit et al., 2005), the resting state EEG of children from different countries might differ due to genetic reasons.

This study was designed with the aim of examining whether healthy children of two strongly differing cultures demonstrate different EEG resting state activity. EEG resting state activity is an energetically costly condition, measured during task-free situations. It is often associated with psychological processes such as mind wandering or self-referenced thoughts. It is also a very stable neurophysiological state, which is strongly genetically determined (Smit et al., 2005; Enoch et al., 2008), and demonstrates considerable stability over long periods of time, leading some authors to suggest that resting state EEG could be used as an individual biological signature (Näpflin et al., 2007). Although resting state EEG is a stable biomarker, it nevertheless depends on expertise (Klein et al., 2016), intelligence (Langer et al., 2012), specific abilities (Jäncke and Langer, 2011), psychopathology (Kindler et al., 2011), personality traits (Knyazev, 2012a), and attachment styles (Verbeke et al., 2014).

Here, we compare the resting state EEG recordings from children aged 10–11 years, living in substantially different cultures, namely Switzerland, as a representative country of the secular Western world, and Saudi Arabia, as a representative non-secular Arabic country. These cultural environments strongly differ in terms of beliefs, practices, and rules, substantially affecting the lives of the children living there, and in addition, the two schooling systems are different. Saudi Arabian children start schooling at the age of six, and their curriculum consists of Arabic language, art education, geography, history, mathematics, Islamic studies and science. Upon completing 6 years, pupils must pass an examination to receive the *General Elementary School Certificate*, which qualifies the student for intermediate school. In the German-speaking region of Switzerland, the children mostly start school at the age of 7, with a curriculum comprising of the German and English languages, art education, geography, history, mathematics and a bit of science. Besides these cultural differences, there are also genetic differences that might exert an influence. The question is whether these different cultural, educational, and genetic backgrounds might influence the wiring and neurophysiology of the children’s brains, which might then influence the resting state EEG. The resting state EEG is used in this study as a marker of general brain activity and brain maturation. With the current state of research, we cannot formulate an explicit hypothesis, as we actually have no clue whether different cultural influences might influence resting state EEG, therefore, this study is designed as a pilot study. Nevertheless, as a first step in identifying neurophysiological differences between populations from strongly differing cultural environments, we consider it worthwhile and interesting to conduct this pilot study and to identify any possible differences.

## Materials and Methods

### Subjects

Ninety-five healthy Swiss children (43 girls, mean age 10.03, range 7–12, SD = 1.6) and 102 Saudi Arabian children (68 girls, mean age 9.95, range 6–13, SD = 1.3) participated in our study. The Swiss children were age matched to the Saudi Arabian children, with no significant differences in age, when analyzed by the Mann-Whitney test. All Saudi Arabian children were born and raised in Saudi Arabia by Saudi Arabian parents and attended Saudi Arabian schools. Similarly, the Swiss children were born and raised in Switzerland and their parents are Swiss. The Swiss and Saudi Arabian children were attending local primary schools. All children were of at least average intelligence and cognitive ability, as evaluated by their teachers and parents (standard IQ tests were not performed since the IQ testing was not part of the original testing procedure approved by the local ethics committees). SES was estimated using criteria adapted from Harmony et al. (1990). Similarly as in Harmony et al. (1990) we used: (a) the level of education of the mother; and (b) income per family member, as measures for classifying SES. For parental “level of education,” the following class categories were used: illiterate, finished first three elementary grades, or finished elementary/secondary/technical school or university. For “income per head,” three classes were also used: below 25% of the minimum monthly wage (low income), between 25 and 50% of the monthly wage (medium income), more than 50% of the minimum monthly wage (high income). For the Swiss sample, 95% of the mothers had at least finished elementary/secondary/technical school. With respect to “income per head,” only 5% had were low income, while 65% were medium, and 30% were high income. Combining these measures according to the criteria of Harmony et al. (1990) into a three-class ranking of SES, 5% of the Swiss sample was classified as low, 35% as intermediate and 60% as good. The Saudi Arabian sample was approximately similar in terms of SES (5% low, 45% intermediate, and 50% good). We performed a cross-tabulation analysis for the SES classification frequencies in order to evaluate whether the SES frequency distribution was similar in Swiss and Saudi Arabian children. This analysis revealed that there was no difference in SES frequency distribution between the two groups (*χ*^2^ = 2.1591, df = 2, *p*-value = 0.3397). All children were free of any neurological and psychiatric diseases. The Saudi Arabian children were attending public schools where they carefully monitor the children for psychiatric and neurological disorders. Similarly, for the Swiss children no psychiatric and neurological disorders have been reported. All children were also explicitly checked for learning disabilities (LD) and attention-deficit hyperactivity disorders (ADHD), and none of the children included in this study suffered from LD and/or ADHD.

All procedures were carried out in accordance with the Helsinki Declaration (1974). All subjects and parents gave informed consent after the procedures had been fully explained to them. Local ethics committees (University Zurich, University Jeddah, and the local school inspectorate of Chur/Switzerland) approved this study.

### EEG Registration

EEG recording of the Saudi Arabian children was accomplished using the 19-channel BEE Medic × 23 system (BEE Medic GmbH). For the EEG recording of the Swiss children, a Mitsar 21-channel EEG system (Mitsar, Ltd., Russia^1^ was used. EEG recording was done according to the protocol presented in Ponomarev et al. (2014). We will briefly reiterate the standard description of the EEG acquisition and pre-processing protocol given in their article. For both groups, we used 19 silver-chloride scalp electrodes that were positioned, according to the international 10-20 system, at sites Fp1, Fp2, F7, F3, Fz, F4, F8, T3, C3, Cz, C4, T4, T5, P3, Pz, P4, T6, O1, and O2. The electrodes were fixed to the scalp using ElectroCaps^2^. The input EEG signals were referenced to linked ears, filtered between 0.5 and 50 Hz and digitized at a rate of 250 Hz. The ground electrode was placed on the forehead. All electrode impedances were kept below 10–5 kΩ. EEG was recorded in eyes closed (EC) and eyes open (EO) resting conditions, for at least 3 min for every period. Subjects were asked to sit still, were instructed not to blink or move their eyes, and let their mind wander. For artifact-correction and pre-processing, WinEEG software was used.

### EEG Data Filtering

Known artifacts were corrected by zeroing the activation curves of individual independent component analysis (ICA; Vigário, 1997; Vigário et al., 2000). We also used templates of eye blink and muscle artifacts for filtering. In addition, epochs with excessive amplitude of filtered EEG and/or excessive faster and/or slower frequency activity were automatically marked and excluded from further analysis. The exclusion thresholds were set as follows, according to previous articles from our group (Ponomarev et al., 2014; Kropotov and Ponomarev, 2015): (a) 100 μV for non-filtered EEG; (b) 50 μV for slow waves in the 0–1 Hz band; and (c) 20 μV for fast waves filtered in the 20–35 Hz band. Finally, the EEG was manually inspected to verify artifact removal. For EEG data analysis, not less than eight artifact-free EEG epochs were used (around 40 s). There was no between-group difference in terms of artifact contaminated rejected EEG epochs (*p* > 0.4). We also tested whether both groups differed with respect to the number of excluded EEG samples and we did not find a Before further processing, the entire array of EEG recordings was filtered at the 2–30 Hz frequency band to minimize the overlearning problem in the ICA algorithm (Vigário, 1997).

### Spectral Analysis

Spectral analysis was performed for the raw (linked ears reference and common average montages) EEG recordings. For each individual, each electrode position and condition power spectrum were computed as described in the following section. Artifact-free continuous EEG was divided into 4.096 s epochs using a Hanning time window (epochs overlapped by 50%) and submitted to Fast Fourier Transform (FFT). Power spectra with a number of averaged epochs less than 10 were eliminated from further analysis. The grand average power spectra were computed for each EEG channel, for each group (Swiss and Saudi Arabian children) and for each condition separately. The absolute power was computed for the delta (2–4 Hz), theta (4–8 Hz), alpha (8–13 Hz), and beta (13–30 Hz) frequency bands, and this was log-transformed for normalization before further statistical analysis.

### Group Independent Component Analysis (gICA)

In order to allow more precise description of spectral differences and to identify the intracortical sources, we conducted a group independent component analysis (gICA). This technique has been used in several articles by our group and has been described in detail in these articles (Ponomarev et al., 2014; Jäncke and Alahmadi, 2016). This technique is roughly similar to the group ICA used in the fMRI research (Calhoun et al., 2001). Thus, we only briefly sketch the basic principles of this technique, as follows. The simplest mixture model, *X(t)* = *AS(t)*, is assumed in the case of ICA, where the output *X(t)* is the *n* × 1 vector of measured potentials (*n* = number of electrodes) at time point *t* (*t* = 1, …, *T*), *A* is the *n* × *n* mixing matrix (where the columns in the A matrix are the independent components (ICs) topographies) and *S(t)* is the *n* × 1 vector of ICs. If A is invertible, then the sources S(t) can be estimated as *S(t)* = *W X(t)*, where *W* = *A* − 1 is the unmixing matrix. The InfoMax algorithm was used in order to obtain estimates of the unmixing matrix *W*. We used a C++ implementation of the Infomax algorithm, which is part of the WinEEG software. This C++ implementation of the Infomax algorithm was practically identical to the runica procedure from the EEGLAB package (Delorme and Makeig, 2004), but with two simple changes. The stopping weight change was reduced from 10^−6^ (default value) to 10^−7^, and the maximum number of iterations was increased from 512 (default value) to 3000. These minor changes allowed the algorithm to work steadily in processing EEG recordings of varying durations, from 40 s to several minutes. The whole set of individual EEG recordings from all children (excluding the epochs containing artifacts) was concatenated into one combined time series, which was then used for assessment of the *W* matrix. The corresponding matrix A was calculated as the inverse of *W*, or *A* = *W*−1. The signals for each individual EEG recording were computed as *S(t)* = *W*×(t). Following the recommendations given in our earlier published article (Ponomarev et al., 2014), the number of signals (components) was chosen to be equal to the number of electrodes (i.e., 19).

### Estimation of Intracortical Sources

For the estimation of intracortical sources of the independent latent components, we used the free LORETA (low-resolution brain electromagnetic tomography) software, provided by The Key Institute for Brain-Mind Research in Zürich, Switzerland^3^. This method is very popular and is frequently used worldwide. A detailed description of this method can be found in the publications of Pascual-Marqui (1999, 2002). A major property of LORETA is that it generates images of current density with exact localization, albeit with low spatial resolution. The low spatial resolution is due to the fact that neighboring neuronal sources are highly correlated. In the LORETA implementation used (there are currently several variants available, eLORETA, sLORETA), the solution space was restricted to the cortical gray matter corresponding to 2394 voxels at a spatial resolution of 7 mm. The Montreal Neurologic Institute average MRI brain (MNI152) was used as a realistic head model for which the lead field was computed. Many articles demonstrating the validity of the LORETA technique have been published. We are entirely aware of the fact that the validity of the sLORETA estimation depends on the number of electrodes used, with more electrodes (mostly) providing better estimations (Michel et al., 2004). However, we would like to point out that we explicitly used a 19-electrode EEG montage here in order to make the EEG registration as nonintrusive as possible for the children. In addition, we were not interested in a perfect intracortical solution; rather, we were interested in obtaining more or less rough imaging of the generators of independent latent components. Nevertheless, we would like to emphasize that intracortical estimations on the basis of 19-electrode EEGs can indeed provide valid results, as has been shown in several studies (Zumsteg et al., 2005a,b, 2006a,b; Thatcher et al., 2014; Emory et al., 2015), especially when using clean EEG data without artifacts. In the current study, we estimated the intracortical sources on the basis of mean ICs, thus diminishing the influence of artifacts.

### Theta/beta Ratio

The theta/beta ratio (TBR) was computed according to Monastra et al. (1999) at Cz where theta is defined as the average EEG power in frequency band from 4 Hz to 8 Hz, and beta is defined as the average EEG power in frequency band from 13 Hz to 21 Hz. The ratio was computed separately for each subject in the EO condition. This ratio was used because several studies have shown that it reflects cortical-subcortical interactions (Knyazev, 2007), and is related to the control of emotional states, dispositional affective traits and emotion regulation (Schutter and Van Honk, 2005; Velikova et al., 2010; Massar et al., 2012; Morillas-Romero et al., 2015).

### Statistical Analysis

For the statistical analysis we adopted the following steps:

1. We focused on the EO condition because this condition is often used in the context of studies examining the diagnostic properties of Quantitative EEG (QEEG) for diagnosing and classifying ADHD and depression. Especially our studies have shown that the diagnostic power of QEEG in the EO condition was superior to that of EC condition simply because in the majority of subjects strong occipital alpha rhythms dominated the spectra in the EC condition and camouflaged other alpha rhythms (Grin-Yatsenko et al., 2009; Ogrim et al., 2012; Ponomarev et al., 2014).
2. For each frequency band (delta: 2–4 Hz, theta: 4–8 Hz, alpha: 8–13 Hz, beta: 13–30 Hz) and electrode position, we performed *t*-tests (transformed using natural logarithm for normalization) to compare the Swiss and Saudi Arabian children, followed by Bonferroni-Holm correction (Holm, 1979). We used a *p*-value of 0.05 (0.05/76 = 0.00065; four bands, 19 electrode positions) to identify statistically significant differences. In addition, we also searched for trends with a *p*-value of 0.10 (0.10/76 = 0.0013). For all tests, we used a two-sided test problem.
3. Since we had to take into consideration the fact that *p*-values depend on sample size, we also calculated effect sizes according to Cohen (1988). Here, the *d*-value was used, which is the difference between two means divided by the accompanying standard deviation. A *d*-value > 0.5 is considered as being moderate, while a *d*-value > 0.8 is considered as being large (Cohen, 1988).
4. In order to identify the intracortical sources of the identified between-group differences, we computed the 19 group-ICs (equaling the number of electrodes used), with each gIC demonstrating a particular topography with maxima at particular electrode positions (Ponomarev et al., 2014). Each component is characterized by a particular spectrographic pattern across the entire frequency spectrum. Thus, there is, for example, a gIC with a maximum at Fz. This gIC is then denoted as cFz (component at Fz). As mentioned above, there are 19 gICs in total, with maxima at each of the 19 electrode positions. A specific advantage of these gICs is that they are statistically independent from one another, which offers several advantages for further analyses (e.g., increased power), and less contamination with artifacts. Here, we will describe only those gICs for which the above-mentioned statistical tests for spectral values revealed a significant result. Thus, when finding a significant between-group difference for Fz, we will present the results for the cFz component and the associated intracortical sources, on the basis of the LORETA software results (Pascual-Marqui, 2002).

## Results

The Swiss and Saudi Arabian children were similar in terms of SES (*p* > 0.3), and age (*p* = 0.5). However, the frequencies for girls and boys were different for both groups (*χ*^2^ = 8.3114, df = 1, *p* = 0.00394). Therefore, we tested whether the power values were different between boys and girls using Hotelling‘s multivariate *t*-tests, with gender as the independent variable, and the power values of each electrode as the dependent variable. These analyses revealed no significant gender difference (Swiss children; EC: *F*_wilks__(76,15)_ = 1.7, *p* = 0.173; EO: *F*_wilks__(76,15)_ = 1.73, *p* = 0.393; Saudi Arabian children: EC: *F*_wilks__(76,15)_ = 1.74, *p* = 0.112; EO: *F*_wilks__(76,15)_ = 1.17, *p* = 0.331). Therefore, we conclude that there is no strong gender difference among our study populations. All the children were healthy, did not suffer from neurological or psychiatric diseases, and demonstrated normal and average academic performance. The TBR was also similar between the children of each culture (*p* > 0.4).

**Figure 1:**
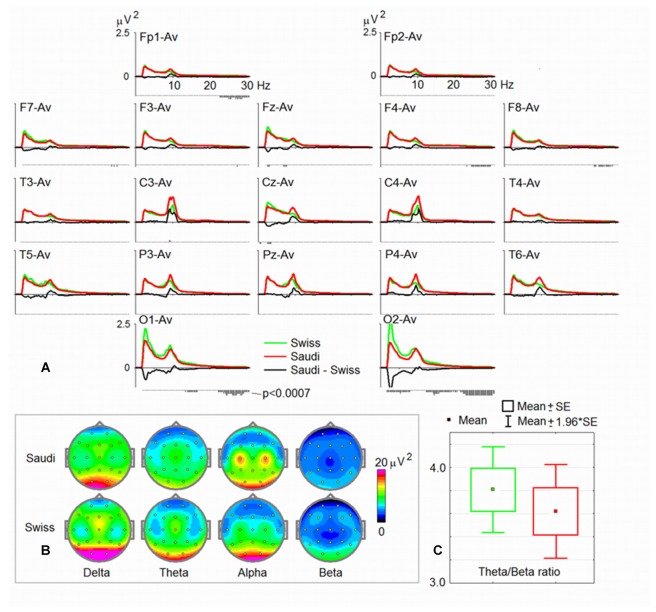
**Spectral characteristics of the two groups. (A)** Electroencephalogram (EEG) power spectra for the resting state with eyes open (EO) in the common average montage in the group of Swiss children (green) and the group of Saudi Arabian children (red), with the difference spectra (Saudi Arabian-Swiss in black). Below each curve, are *p*-values of statistical significance (the lowest vertical bar corresponds to *p* < 0.0007). **(B)** Maps of EEG spectra for four band ranges (delta, theta, alpha and beta). **(C)** The mean of the theta/beta ratio (TBR) for the two groups.

Figure 1A shows the average spectral curves for the Swiss and Saudi Arabian children. The same figure also demonstrates the average topoplots (Figure 1B) for the Swiss and Saudi Arabian children, broken down into the four frequency bands. In addition, a box plot is shown for the TBR. In Table 1, the *t* and *p*-values, together with the accompanying Cohen’s *d*-values, are shown for each electrode position and frequency band.

**Table 1:** **Differences between spectra obtained during the *Eyes Open* (EO) condition**.

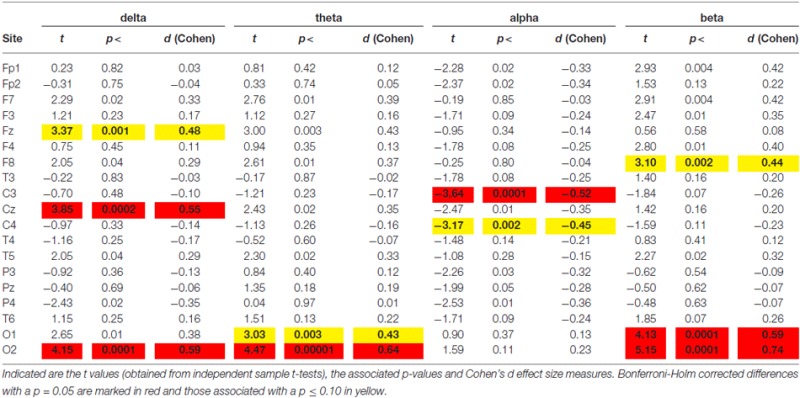

As can be seen from Figure 1C (strongly overlapping distributions), there is no difference between the Swiss and the Saudi Arabian children’s TBR. The ratios for both groups are also within the normal range, and substantially differ from ratios found for children diagnosed with ADHD, who demonstrate substantially larger TBRs, when using the HBImed database of normal and healthy children of the same age. However, when referring to previous publications reporting TBRs (Ogrim et al., 2012; Arns et al., 2013) from ADHD children, the ratios of our sample are considerably smaller.

As can be seen from Figures 1A,B and Table 1, the Saudi Arabian children demonstrated stronger alpha band power at central electrode positions (C3 and C4). For C3, the between-group difference was significant (see Table 1), even after Bonferroni-Holm correction. For C4, there is a trend toward a between-group difference (*p* = 0.002 uncorrected, Cohen’s *d-value* = 0.45). The Swiss children demonstrated stronger power at the occipital electrode positions (for delta, theta, and beta). They also showed stronger delta band power at Fz. For Fz, the Swiss children demonstrated elevated power values for the theta band (*p* = 0.003, not corrected for multiple comparison, Cohen’s *d* = 0.43).

**Figure 2:**
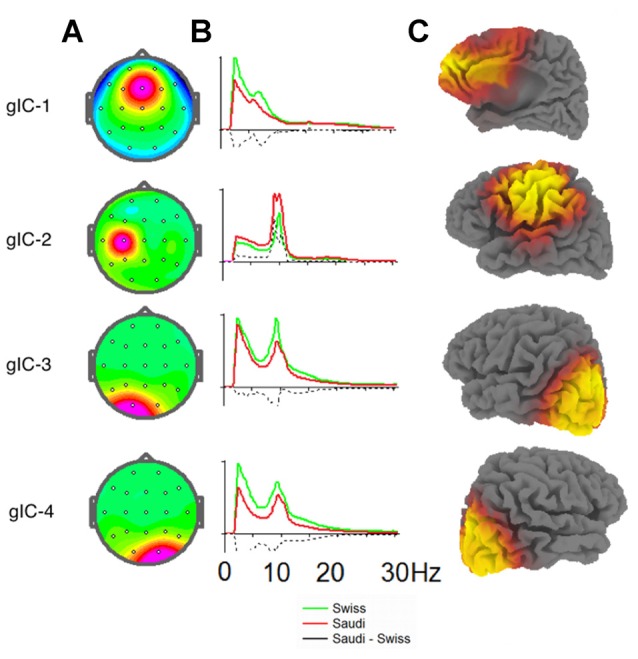
**(A)** Topographies of the four group independent components (gICs) discriminating best between the two Swiss and Saudi Arabian children for the EO condition.** (B)** EEG spectra in standard units for the ICs of both groups. Spectra for the Swiss children are shown in green, spectra for the Saudi Arabian children are shown in red. Differences between the Swiss and Saudi Arabian children are indicated by dashed black lines. **(C)** sLORETA images separately for each depicted IC. The strength of the intracortical sources are indicated as heat maps on the sLORETA images, with yellow showing the brain regions with the strongest intracortical sources in current density.

The four ICs for the electrode positions demonstrating the above-mentioned between-group differences are shown in Figure 2. This figure also shows the intracortical sources of these differences. Maximum current density of the cFz component (where Swiss children showed the strongest power, especially in the delta band) was found in the mesial anterior frontal cortex, comprising the anterior cingulum and the mesial part of the middle frontal gyrus. However, the identified current densities were also found at the entire mesial wall of the frontal cortex, the dorsal frontal cortex, supplementary motor cortex, ventral cingulum and the ventro-mesial frontal cortex.

The intracortical sources for the enhanced alpha band power at C3 for the Saudi Arabian children were located in the left sensory-motor cortex, extending into the inferior parietal lobule and into the ventral frontal, as well as dorsal lateral frontal cortices. The intracortical sources for the enhanced power values in the delta, theta, and beta bands for the Swiss children were located bilaterally in the occipital cortex and extended slightly into the inferior posterior temporal cortex.

## Discussion

We conducted this pilot study in order to compare EEG resting state measures in children from two different cultural settings. Our hypothesis was that if children are exposed to different cultural influences associated with different beliefs, rules and behavioral practices, it might have an impact on the wiring and neurophysiology of the children’s brains. The different cultural contexts might also influence their style of thinking and cognitive behavior in a resting state situation. A further possibility we took into consideration was the possible genetic difference between children from Saudi Arabia and children from Switzerland, which might influence resting state EEG measures.

In fact, we found substantial similarities between children from Saudi Arabia and Switzerland with respect to resting state EEG activity. This similarity is striking, since the two groups had been raised in very different cultures. However, there were also some slight, but nevertheless interesting, differences. There were actually three substantial differences, which will be discussed in the following section, namely the different activation over central electrodes in the alpha band, increased power over occipital electrodes in the delta, theta, and beta bands, and increased delta power at *Fz*.

Swiss children demonstrated stronger power in the delta band at *Fz* than the Saudi Arabian children. The intracortical sources of these power increases are mostly located in the medial wall of the frontal cortex, comprising the anterior cingulum, dorsal cortex, and middle frontal cortex. These brain regions are known to be strongly involved in the control of executive functions. A stronger delta band in children at the age of those examined in our study, is often associated with delayed maturation; thus, one might argue that the Swiss children demonstrated a less mature brain than the Saudi Arabian children. However, other possible reasons for this delta band enhancement should also be considered. Delta band oscillations are prominent in early developmental stages, during slow-wave sleep, and are often identified in the context of developmental disorders and pathological conditions. Recently, Knyazev (2012b) suggested an association between delta band oscillations and autonomic, as well as metabolic, processes. Based on this assumption, he argues that delta band oscillations might be involved in the integration of cerebral activity with homeostatic processes, which may be involved in the control of motivation. Several studies have shown that delta band power increases during hunger, sexual arousal, panic attacks, sustained pain and substance abuse. Whether any of these issues are relevant to the excess frontal delta band power in Swiss children is difficult, or impossible, to determine on the basis of current knowledge. A note on possible genetic influences is necessary here. In the seminal study of Smit et al. (2005), delta band activity during resting state was associated with a moderate heritability score, which was, however, the lowest heritability score compared to all other frequency bands (*h*^2^ = 0.62).

As a second finding of our study, Saudi Arabian children demonstrated stronger alpha band power at *C3* compared to Swiss children. Referring to the cC3 component from the group ICA, and the associated LORETA image, it is obvious that the entire sensorimotor region, comprising the motor cortex, the sensorimotor cortex, and parts of the inferior parietal lobule, as well as the inferior frontal gyrus, are involved here. Thus, Saudi Arabian children demonstrated stronger alpha band activity in a relatively large region comprising areas involved in the control of different psychological functions (e.g., attention, spatial processing, motor control, etc.). In addition, some of these brain regions are part of the mirror neuron system (e.g., left inferior frontal gyrus and left inferior parietal lobule).

The topography and peak frequency of this power increase in the alpha band roughly resembles the so-called mu- (9–12 Hz) and/or sensorimotor rhythm (SMR, 12–15 Hz). The meaning of SMR and mu-rhythms are not entirely clear so far. SMR and mu-rhythms appear primarily during states of immobility while the sensorimotor areas are either idling or inhibited. SMR and mu disappear or decrease when the sensorimotor areas are becoming active, which typically happens during the performance of motor tasks or when the subjects imagine moving. The mu wave is even suppressed when one observes another person performing a motor action. Therefore, it has been suggested that mu-rhythm suppression indicates the involvement of the mirror neuron system (Oberman et al., 2005) and other cognitive functions (Vernon et al., 2003). For example, manipulating SMR in the context of neurofeedback training is often associated with improved attention, executive functioning, and creativity (Gruzelier, 2014; Gruzelier et al., 2014), as well as improvements in subjects suffering from learning difficulties (Tansey, 1984, 1993), ADHD (Vernon et al., 2003) and substance abuse issues (Unterrainer et al., 2014).

Alpha band oscillations are prominent during psychological states, which do not require attention to the environment (Cooper et al., 2003; Palva and Palva, 2007). There is a growing consensus that increased alpha power is associated with active inhibition of sensory networks or brain networks that are not needed for the control of on-going tasks, but rather for simultaneous maintenance of alertness (Coste et al., 2011). Thus, increased alpha band power in these brain regions during resting state in the Saudi Arabian children most probably indicates a stronger inhibition, making the children more relaxed and supporting a more strongly inward direction of attention.

It should be noted here that alpha band oscillations are typically strong during resting state conditions. During resting state, the subjects generally let their minds wander, or are engaged in some kind of self-referential thoughts. It is possible that the Saudi Arabian children perform more mind-wandering or self-referential thinking than Swiss children. It might also be that their more intensively practiced religious techniques and rituals support a deeper and more intensive self-referential thinking style.

Currently, only a few studies have examined intercultural differences in cortical resting state measures. For example, Knyazev et al. (2012) compared subjects from a more Western culture (Russia) with subjects from a more Eastern culture (Taiwan) and registered EEG oscillations during self-referential thoughts. They identified that in Russian subjects, spontaneous self-referential processes were accompanied by enhanced alpha oscillations in posterior brain regions (posterior parts of the default mode network), whereas in people from Taiwan, self-referential processes were accompanied by enhanced alpha in frontal brain regions (anterior parts of the default mode network). Thus, different cultural backgrounds are associated with different patterns of alpha band oscillations. Of interest, two other studies found that adult Muslim prayer givers demonstrate enhanced alpha band power during their *salat*, especially at the parietal and occipital regions (Doufesh et al., 2012, 2014), which are not the same as the regions identified for our Saudi Arabian children. However, these studies demonstrate that people from Muslim communities exhibit specific alpha band features.

Resting state alpha band activity has been shown to be strongly heritable in adults, with an average *h^2^* = 0.90 across all electrodes and an *h*^2^ = 0.92 at *C3* (Smit et al., 2005). Thus, the possibility that genetic influences might be responsible for the elevated alpha band activity at C3 in Saudi Arabian children should be considered seriously.

Finally, we identified stronger power values in Swiss children for all frequency bands (except for the alpha band) at occipital electrode positions (*O1* and *O2*). The intracortical sources of the gICs at *cO1* and *cO2* were located bilaterally in the occipital cortex, extending into the inferior temporal cortex. The stronger power in these brain areas is difficult to explain, mainly because three out of four frequency bands demonstrated stronger power in Swiss children. In addition, these frequency bands have not been shown to be involved in the psychological functions typically associated with the occipital cortex. However, it is known that power values of different frequency bands are not independent of one other. In some studies, the correlations between frequency bands are relatively strong (de Munck et al., 2009; Jäncke et al., 2015). The stronger power values might indicate a differential functioning of the neural networks located in the occipitotemporal region. It could be that these neurons oscillate more synchronously in Swiss than in Saudi Arabian children. In any case, the reason for this striking between-culture difference is difficult to explain. One possibility could be that people in Saudi Arabia focus more strongly on auditory than visual information. For example, they generally prefer listening to stories to reading text. For example, Nuzhat et al. (2011) reported that the auditory learning mode is the most preferred learning mode among medical students from Saudi Arabia. Whether the auditory learning mode is also the most preferred in Saudi Arabian children at the age of 10 remains to be demonstrated. However, if students from Saudi Arabia are indeed more inclined to use auditory information for learning compared to Western students, it might result in weaker and less frequent visual input, which could in turn affect the neural wiring of the visual system and its adjacent brain regions.

Taken together, the results of this pilot study have shown that many similarities exist with respect to EEG resting state features of children from two different cultures. Beside these similarities, some relatively strong differences have also been uncovered; these are stronger delta band power values in the mesial frontal regions and stronger power values in three out of four frequency bands in the occipital areas in Swiss children. For Saudi Arabian children we uncovered stronger alpha band power over the sensorimotor cortex. Although we have discussed some possible reasons (educational and environmental) for these differences, it is currently unclear what actually causes these differences.

As a possible alternative influence, we must take genetic differences into account. Several studies have shown that in adult subjects, EEG power at rest is a heritable trait across the entire frequency spectrum (Smit et al., 2005; Enoch et al., 2008), with the alpha and theta bands being the most heritable frequency bands. In our study, we identified locally focused between-group differences at either *cFz* or *cC3*, or *cO1* and *cO2*. The differences were found in either one particular frequency band per electrode position (delta for *Fz* and alpha for *C3*), or in several frequency bands (delta, theta and beta for *O1* and *O2*). Thus, if genetic influences are responsible here, they might have exerted very specific and locally restricted influences. So far, it is known that genes influence EEG power through effects on the conductive properties of the tissues surrounding the cortex. For example, skull and scalp thickness (which are most likely heritable traits) strongly influence EEG power measures (Babiloni et al., 1997). Further possible genetic sources for EEG characteristics may be found for cerebral rhythm generators (e.g., hippocampal slow-wave activity or the thalamocortical and corticocortical generators of cortical alpha Lopes da Silva, 1991), the number of pyramidal cells, the number of dendritic connections, or their orientation with respect to the scalp (Ray, 1990). However, since we found locally restricted differences, the possible above-mentioned reasons for genetically driven between-group differences in EEG power might also be locally restricted. Whether locally restricted anatomical differences between Saudi Arabian and Swiss children really exist should be investigated in future experiments.

A special note is warranted for the TBR used in this study, and for which we did not find a between-group difference. Currently, there is much discussion on whether or not this ratio can be used as a biomarker for diagnosing ADHD in children (Arns et al., 2013; Loo et al., 2013; Snyder et al., 2015). The conclusions as to whether this is indeed a valid biomarker are mixed, however, there is evidence that this ratio is nevertheless a useful addendum for ADHD diagnosis when it is applied in addition to classical psychiatric diagnostic techniques (Boutros et al., 2005; Snyder et al., 2015). More importantly for the scope of the present article, are those articles demonstrating that the TBR is related to cortical-subcortical interactions associated with emotion regulation, attentional and/or cognitive control (Schutter and Van Honk, 2005; Knyazev, 2007; Putman et al., 2010; Massar et al., 2012; Morillas-Romero et al., 2015). Based on our findings, we can clearly state that Swiss and Saudi Arabian children do not differ in this biomarker, and thus we can conclude that the neurophysiological and psychological mechanisms, which are associated with this ratio are similar for these children. Therefore, basic cortical-subcortical interactions are obviously similar in children coming from strongly differing societies.

We have discussed the reported neurophysiological differences between Swiss and Saudi Arabian children in the context of different cultural and possible genetic influences. Which of these influences are most relevant here are difficult to disentangle on the basis of our data. We know from genetic studies that people from European and Arabian cultures demonstrate at least subtle genetic differences (Li et al., 2008). In addition, it has been reported that several diseases with genetic origins are more frequently occurring in Arabian societies (Tadmouri et al., 2014). Whether these or other genetic influences might have influenced brain anatomy and brain function is (to the best of our knowledge) not known so far. Thus, our above-mentioned argumentations with respect to possible genetic influences are still speculative. However, it would be interesting and worthwhile to relate neurophysiological and neuroanatomical measures to particular genetic characteristics. For this it will be necessary to conduct a large-scale intercultural genetic study in combination with neuroanatomical and neurophysiological examinations. But this kind of study has to be done in the future.

Nevertheless, these findings are of some importance for those who are interested in QEEG data, and who use databases to refer their own results to standard databases. As shown by our study, researchers must be careful when comparing their own EEG data with database data from a different culture, since that could lead to misinterpretations. Thus, it is necessary to design QEEG databases that not only fit the age, gender and SES of the examined subjects, but also their culture. Apart from these practical issues, this study emphasizes the necessity of studying possible between-culture differences with respect to neurophysiological and neuroanatomical features. From these potential differences, we can learn a great deal about brain plasticity induced by experience.

## Author Contributions

NA carried out the study in Saudi Arabia, supervised data acquisition, designed the study and participated in drafting the manuscript. LJ designed the study, participated in data analysis and drafted the manuscript. SAE and YK supervised data analysis and participated in drafting the manuscript. AMM was responsible for recording EEG data in Chur/Switzerland and participated in drafting the manuscript.

## Conflict of Interest Statement

The authors declare that the research was conducted in the absence of any commercial or financial relationships that could be construed as a potential conflict of interest.

## Footnotes

^1^http://www.mitsar-medical.com

^2^http://www.hbimed.com/de/qeeg/shop/qeeg-systeme/electro-cap-set-ii.html

^3^http://www.uzh.ch/keyinst/loreta.htm
